# The Influence of Social Support on Quality of Life of Men Who Have Sex with Men in China: A Preliminary Study

**DOI:** 10.1371/journal.pone.0127644

**Published:** 2015-05-26

**Authors:** Jie Liu, Bo Qu, Yaxin Zhu, Bingxue Hu

**Affiliations:** Department of Health Statistics, School of Public Health, China Medical University, Shenyang, Liaoning Province, China; Fudan University, CHINA

## Abstract

The quality of life (QOL) of men who have sex with men (MSM) has received increasing attention in recent years. Our study surveyed the QOL and explored the influence of social support on QOL in Chinese MSM. A cross-sectional survey was conducted from September 2013 to March 2014 of 438 MSM in Huludao and Zhengzhou City, China. The results of univariate analysis showed that higher QOL scores were associated with receiving psychosocial counseling, higher health education, younger age and marital status of being single or unmarried *p* < 0.05). The structural equation model fitted well, with χ^2^ = 2083.47 (*p* < 0.05), RMSEA = 0.07, and GFI = 0.88. Among the latent factors, social support, with a factor load of 0.47, had greater impact on QOL than demographic characteristics. Within social support, the item loads for psychosocial counseling and health education were 0.17 and 0.29, respectively. Basic demographic characteristics also influenced social support, with a factor load of -0.65. For demographic characteristics, the greatest item loads were for marital status and age (0.77 and 0.71, respectively). These findings suggest that strengthening social support, especially for older and married individuals, would improve QOL in MSM in China.

## Introduction

In China, the HIV epidemic among Chinese men who have sex with men (MSM) continues to expand rapidly [1]. Sentinel surveillance data have shown that the prevalence of HIV in Chinese MSM increased from 2.0% in 2007 to 6.3% in 2011 [2]. The increase in the percentage of new HIV cases is still dramatic. Chinese MSM accounted for 0.2% of new infections in 2001, 12.2% in 2007, and 32.5% in 2009 [3,4]. The MSM population has become a high risk group for sexually transmitted disease (STD) infection in China due to their high number of partners, unprotected anal intercourse (UAI) and high migration rates [5–7]. MSM may also play a bridging role in the spread of HIV and other STDS from a high-risk population to the general population, which presents a concentrated public health problem [8]. In China, MSM have recently gained more attention from the Chinese Government in terms of HIV prevention and care [9].

The health-related quality of life (HRQoL) has increasingly been acknowledged as an important and valid health outcome measure in health services research, clinical trials, and evaluation [10]. Bucciardini et al. also proposed that the HRQoL could be an effective measure of health status [11]. Quality of life (QOL), especially mental health, can affect HIV-related sexual risk behavior in MSM, and, therefore, there is a need for comprehensive information about the health status of the MSM population [12,13]. Furthermore, mental HRQoL negatively associates with abuse, violence and HIV-related high-risk behaviour among homosexual and bisexual MSM [14]. Identification as gay or homosexual is related to poorer mental health [15,16]. The MSM population is also more concerned about their physical health than men from the general population [17]. Studies of QOL may help to understand the mental and physical status of MSM and to identify influencing factors to improve HIV prevention efficacy [12,17].

Many studies have investigated HRQoL in HIV-positive patients and people living with HIV/AIDS (PLWHA) [10,18–22]. Some have examined the association between HRQoL and different risk factors. Razavi et al. showed that, of PLWHA, participants older than 35 years and unemployed participants had significantly lower scores in overall quality of life [23]. Several studies have found that social support is a major factor positively affecting HRQoL in general and HIV-infected populations [20–22,24–26]. A previous study suggested that HIV counseling and testing could promote safe sexual behavior in PLWHA [21]. However, limited information is available about the relationship between QOL and social support in the Chinese MSM population.

In the present study, our aim was to survey the QOL of Chinese MSM and examine the relationship between QOL and social support in order to provide more effective HIV prevention information.

## Materials and Methods

### Ethic statements

Participation was voluntary and verbal informed consent was obtained from each of the participants prior to interview. To maintain privacy and anonymity, no personal identifying information or written consent were collected. This research was approved by the Bioethics Advisory Commission of China Medical University.

### Respondents and procedures

A cross-sectional study was conducted from August 2013 to March 2014 in two Chinese cities, Zhengzhou and Huludao. Respondents were recruited from the internet, bars and saunas. All potential participants underwent a face-to-face explanation to a standardized questionnaire. All questionnaires were self-administered in a private room using the standardized version of the questionnaire.

### Questionnaires

The Health Survey Short Form (SF-36) questionnaire contained 36 items and measured domains of health-related quality of life with 8 scales: physical functioning (PF), role limitation due to physical problems (RP), bodily pain (BP), general health perceptions (GH), vitality (VT), social functioning (SF), role limitation due to emotional problems (RE), and mental health (MH). Each scale is standardized on a 0–100 metric, with higher scores indicating better functioning. These dimensions were further categorized into physical component summary (PCS) and mental component summary (MCS). PCS was drawn from PF, RP, BP and GH, whereas MCS was drawn from VT, SF, RE and MH. The total score (QOL) of the SF-36 included both physical and mental health statuses. The scores of PCS, MCS and QOL ranged from 0 to 100, with higher scores representing better health [27]. The questionnaire also included demographic and social support sections. Demographic information items included age, marital status, education level, vocation, and monthly income. Social support items included family or friend awareness of sexual orientation, psychosocial counseling, health education, and receiving condoms, lubricants, peer education, HIV/syphilis testing, and AIDS information materials.

### Statistical analysis

In this study, a structure equation model (SEM) was used to measure the relationship between observed and latent variables. The factor loads of the structure equation model ranged from 0 to 1. SEM can show direct and indirect relationships between variables by path maps [28–34]. In addition, whether the relationships are reasonable could be verified based on the results of SEM [28].

The distributions of total scores of the categorical QOL variables were compared by the Student’s *t*-test and one-way ANOVA. Reliability analysis of the data and univariate analyses were performed using SPSS version 16.0 (SPSS Inc., Chicago, IL, USA) for Windows, and the SEM was produced using LISREL 8.5. χ^2^, RMSEA (root mean square error of approximation), SRMR (standardized root mean square residual), GFI (goodness-of-fit index), AGFI (adjusted goodness-of-fit index), and IFI (incremental fit index) were used to indicate whether the model was an adequate fit [34].

## Results

### Participant characteristics

A total of 453 MSM were surveyed for the study and 438 MSM completed the questionnaire (response rate: 96.7%). The ages of respondents ranged from 18 to 74 years, with an average age of 28.26 ± 7.91 years. 186 (42.5%) respondents self-identified as bisexual and 252 (57.5%) as gay. The PCS and QOL scores for older individuals were significantly lower than those for younger individuals (*p* < 0.05). The PCS, MCS and QOL scores in the married group were lower than in the other two groups (*p* < 0.05). Compared to other respondents, the unemployed had the lowest MCS scores (*p* < 0.05). The PCS, MCS and total QOL scores increased with income level (*p* < 0.05). Participant characteristics and demographic information is outlined in Table 1.

**Table 1:** Comparison of SF-36 scores for the MSM population by participant characteristics.

Variable	PCS		MCS		QOL	
	Mean ± SD	F value	Mean ± SD	F value	Mean ± SD	F value
**Age** (years)		15.3		1.1		4.6
≤25	80.5 ± 18.6**		60.7 ± 20.8		70.6 ± 18.2**	
25–35	74.0 ± 18.2		62.6 ± 21.9		68.3 ± 18.4	
35–45	70.8 ± 17.8		60.0 ± 18.3		65.4 ± 20.1	
≥45	60.2 ± 16.4		59.7 ± 17.6		60.0 ± 18.0	
**Marital status**		25.0		10.2		17.7
Single	77.1 ± 21.9**		65.0 ± 19.9**		71.1 ± 20.5**	
Married	59.9 ± 18.8		55.3 ± 16.7		57.6 ± 18.2	
Divorced or widowed	65.4 ± 20.0		56.9 ± 18.4		61.2 ± 18.9	
**Education**		1.2		1.1		1.0
Junior high school or lower	77.1 ± 14.8		59.5 ± 17.6		68.3 ± 17.9	
Senior high school	74.8 ± 18.1		61.2 ± 18.7		68.0 ± 19.1	
College or above	76.8 ± 20.2		62.6 ± 19.5		69.7 ± 19.3	
**Vocation**		1.2		3.0		1.0
Unemployed	72.0 ± 15.9		70.4 ± 14.9*		71.2 ± 19.6	
Student	72.1 ± 16.6		72.8 ± 16.1		72.4 ± 18.7	
Blue collar	72.3 ± 16.8		72.5 ± 16.0		72.4 ± 21.9	
White collar	70.5 ± 17.1		74.9 ± 15.8		72.7 ± 20.9	
**Monthly income**		9.8		9.5		6.2
≤1000 Yuan	71.2 ± 16.7**		58.5 ± 18.6**		64.9 ± 18.4**	
1000–3000 Yuan	72.1 ± 17.2		66.4 ± 20.2		69.2 ± 21.2	
≥3000 Yuan	79.9 ± 18.3		68.6 ± 19.9		74.2 ± 22.4	

PCS, Physical Component Summary; MCS, Mental Component Summary; QOL, quality of life. One-way ANOVA was used for data analysis

*p < 0.05.

** *p* < 0.01.

### Reliability and validity analysis

The Cronbach’s α coefficient for our analysis was 0.96, indicating good reliability. In the validity analysis, the Bartlett’s test was significant (*p* < 0.01), whereas the Kaiser-Meyer-Olkin measure was 0.78, justifying the application of a factor-analytic procedure, and eight factors accounted for 88% of the variance (Table 2).

**Table 2:** Factor analysis results.

SF-36 Item	PF	RP	BP	GH	VT	SF	RE	MH
PF01	0.68							
PF02	0.87							
PF03	0.91							
PF04	0.93							
PF05	0.94							
PF06	0.93							
PF07	0.96							
PF08	0.97							
PF09	0.99							
PF10	0.95							
RP1		0.94						
RP2		0.93						
RP3		0.95						
RP4		0.93						
BP1			0.94					
BP2			0.96					
GH1				0.62				
GH2				0.81				
GH3				0.88				
GH4				0.84				
GH5				0.87				
VT1					0.58			
VT2					0.64			
VT3					0.83			
VT4					0.81			
SF1						0.88		
SF2						0.70		
RE1							0.94	
RE2							0.95	
RE3							0.91	
MH1								0.64
MH2								0.74
MH3								0.55
MH4								0.86
MH5								0.64

PF, physical function; RP, role-physical; BP, bodily pain; GH, general health; VT, vitality; SF, social function; RE, role-emotional; MH, mental health.

### Social support

A total of 66.7%, 64.4% and 56.1% of the MSM surveyed received condoms, lubricant, and peer education, respectively, in the past 12 months. The proportions that received STD testing and informational materials in the past 12 months were 25.5% and 60.8%, respectively. A total of 10.7% and 59.8% of the respondents received psychosocial counseling and attended health education, respectively, in the past 12 months, and 8.6% of the MSM surveyed disclosed their sexual orientation to relatives or friends. In the univariate analyses, respondents who received STD testing had higher physical health scores (*p* < 0.05), and respondents who concealed their sexual orientation from relatives and friends had lower mental health scores (*p* < 0.05). The respondents receiving psychosocial counseling and health education had higher PCS, MCS and QOL scores (*p* < 0.05). These results for social support are summarized in Table 3.

**Table 3:** Comparison of SF-36 scores for the Chinese MSM population by social support received.

Item	PCS		MCS		QOL	
	Mean ± SD	*t value*	Mean ± SD	*t* value	Mean ± SD	*t* value
Received condoms		0.7		0.8		0.7
Yes	71.2 ± 19.8		56.1 ± 16.6		63.6 ± 18.4	
No	69.8 ± 19.1		54.7 ± 17.0		62.2 ± 20.6	
Received lubricants		0.7		0.5		0.5
Yes	71.7 ± 18.6		58.8 ± 16.2		65.3 ± 21.0	
No	70.4 ± 19.9		58.0 ± 15.2		64.2 ± 20.2	
Received peer education		0.9		0.5		0.7
Yes	75.4 ± 19.4		71.9 ± 22.6		73.6 ± 19.6	
No	73.8 ± 19.2		70.8 ± 20.8		72.3 ± 18.1	
Received STD (HIV/syphilis) testing		2.4		0.3		0.9
Yes	80.5 ± 17.2*		68.2 ± 20.6		74.4 ± 19.9	
No	76.1 ± 16.4		69.0 ± 23.4		72.5 ± 20.1	
Received psychosocial counseling		2.3		2.2		2.1
Yes	80.1 ± 15.2*		86.0 ± 18.2*		83.0 ± 16.8*	
No	74.7 ± 15.0		79.9 ± 17.9		77.3 ± 17.4	
Received health education		2.5		4.1		3.4
Yes	75.6 ± 15.2*		80.9 ± 20.0**		78.2± 18.7**	
No	71.7 ± 16.7		72.5 ± 22.9		72.1 ± 18.4	
Received AIDS information materials		0.3		0.5		0.5
Yes	71.6 ± 16.4		76.5 ±19.9		74.1 ± 15.7	
No	71.1 ± 17.2		75.6 ± 16.3		73.3 ± 16.1	
Sexual orientation disclosed to at least one relative or friend		0.5		2.1		1.2
Yes	69.3 ± 15.7		80.7 ± 14.9*		75.0 ± 17.0	
No	68.1 ± 14.6		75.5 ± 14.7		71.8 ± 15.9	

PCS, Physical Component Summary; MCS, Mental Component Summary; QOL, quality of life. Student’s t-test was used for data analysis.

* *p* < 0.05.

** *p* < 0.01.

### Relationships between QOL, social support and demographic characteristics

**Fig 1:**
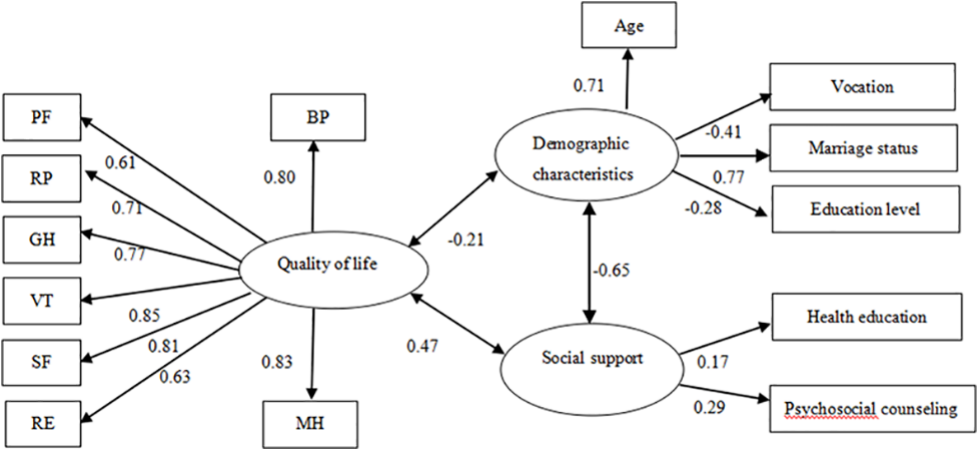
The structural equation modeling of the factors that influence QOL among Chinese MSM. Path diagram illustrating the relationships between demographic and social support factors and QOL in Chinese MSM. Social support had the greatest impact on QOL, and demographic characteristics appeared to influence the likelihood of receiving social support.

To assess the relationships between QOL, social support, and demographic characteristics information, a hypothesized recursive SEM was used to estimate the magnitude and direction of the interdependent effects using the maximum likelihood estimation method. The goodness results of best fit SEM showed χ^2^ = 2083.47 (P<0.00), RMSEA = 0.07, CFI = 0.88, GFI = 0.83, NFI = 0.87, and AGFI = 0.89. We estimated the direct effects of one factor on the others by path coefficients proximal to the unidirectional. The item loads for the eight QOL domains were high (ranging from 0.61 to 0.85). The factor load between QOL and social support was 0.47, which was higher than the load between QOL and demographic characteristics. For social support, the item loads for psychosocial counseling and health education were 0.17 and 0.29, respectively. Demographic characteristics also had influence on social support, with a factor load of -0.65 and, marriage status (0.77) and age (0.71) exhibited greater item loads. The relationships between demographic characteristics and social support are shown in Fig 1.

## Discussion

The results indicated that the SEM fit our data well. For the latent factors, social support had the greatest impact on QOL, and some demographic characteristics appear to influence the likelihood of receiving social support. For the observed variables, the item loads for health education and psychosocial counseling in social support were the highest and for the observed demographic factors, the greatest item loads were seen for marital status and age.

Social support was also related to physical and mental health in general populations in the world and also for individuals with HIV [21,26,35]. Effective social support can improve mental well-being, reduce substance abuse, and promote other positive health behaviors in MSM, whereas individuals who receive less social support are more likely to practice unsafe sex [36].

Homosexuality is not well accepted in China, and therefore MSM often self-isolate for fear of discrimination from society and family, which may lead to depression and decreased QOL [37,38]. Depression is known to negatively impact QOL and to accelerate disease progression in people living with HIV/AIDS [39,40]. Taken together, these findings suggest that social support should be actively implemented to reduce depression and enhance QOL in the Chinese MSM population.

Among the factors included in social support, health education and psychosocial counseling had the greatest impact on QOL. Bastardo and Kimberlin suggested that psychosocial support from professionals and non-professionals is a critical element of HIV care and can help enhance the QOL of patients in developing countries [41]. Social support and counseling to reduce stress have been found to effectively enhance interventions and reduce HIV-related high-risk behaviors in MSM [42,43]. Although the benefits of psychosocial counseling are obvious, only a small portion of the respondents (10.7%) in our study had received this type of support. Enhancing psychosocial counseling for Chinese MSM is an urgent priority that will likely be important in improving health status.

Our results also indicated the importance of health education in the MSM population. Mohamed and Mahfouz found that insufficient public health education for PLWHA resulted in misconceptions that may encourage engaging in risky behavior, such as not using condoms because they are perceived as only a contraceptive rather than a means to prevent STDs [44]. Psychosocial counseling and health education can also help reduce fear of taking the HIV test [45]. Furthermore, both HIV education and psychological counseling contributed to an increase in HIV testing in the MSM population [36]. Our results suggest that vigorously strengthening health education and psychosocial counseling programs could considerably improve QOL of the MSM population in China.

Other aspects of social support also showed great effectiveness in improving QOL in our study. For example, respondents that received STD testing had higher physical health scores. Hoover et al. proposed that increasing STD screening would greatly help to lower the prevalence of STDs in MSM in the United States [46]. Therefore, prevention programs that include STD testing should be implemented to enhance the health of the MSM population. Our study also found that concealment of sexual orientation from family and friends was associated with lower mental health scores, consistent with findings for Swedish MSM [12]. The traditional Chinese concept of filial piety and system of patrilineality may lead MSM to feel that they have failed in their duty to continue the paternal line and that they have disgraced their family, which likely increases stress in MSM and limits their willingness to disclose their sexual orientation to their family [47]. However, receiving emotional support from a family member or a sexual partner was shown to improve mental health in African American MSM [48], suggesting that emotional support from family or friends may improve the mental status of Chinese MSM.

We also found that certain demographic characteristics appeared to influence the likelihood of individuals receiving social support. Specifically, marital status and age strongly negatively correlated with social support. Many married Chinese men may not pursue HIV testing due to concern that their spouse or family members will discover their sexual orientation [49]. Fear of straining marital relationships and the possibilities of abandonment, divorce, or even violence have been identified as barriers for married MSM to pursue HIV testing [50,51]. Additionally, with increase in age, the initiative to seek social support declines [52].

This study has several limitations. Firstly, due to the social stigma of homosexuality in China and the consequent hidden nature of the MSM population, potential respondents may have decided not to participate in our study in order to protect their privacy, decreasing representation from more Chinese MSM. Secondly, the sample size was relatively small and the participants were recruited from only two cities in China and, therefore, may not be representative of all Chinese MSM. However, our study identified multiple factors that may influence QOL in MSM that can be addressed separately and in depth in future research.

## Conclusion

Our study displayed the impact of social support on QOL in Chinese MSM and highlighted the urgent need to strengthen intervention efforts, with more emphasis on social support networks and programs to improve QOL, especially for older and married MSM.
